# Spectrum of paediatric rheumatic disorders at a tertiary hospital in Tanzania

**DOI:** 10.1186/s12969-020-0418-2

**Published:** 2020-04-03

**Authors:** Francis F. Furia, Evance Godfrey, Naomi Mwamanenge, Peter Swai

**Affiliations:** 1 Department of Paediatrics and Child Health, School of Medicine, Muhimbili University of Health and Allied Sciences, Dar es Salaam, Tanzania; 2 Department of Paediatrics and Child Health, Muhimbili National Hospital, Dar es Salaam, Tanzania

**Keywords:** Rheumatic disorders in Tanzania, Paediatric rheumatic disorders, Tanzania

## Abstract

**Background:**

Paediatric rheumatic disorders are common in children and result in significant impairment in quality of life, morbidity and mortality. There is limited information on the burden of these disorders in lower income countries especially in sub-Saharan Africa. Few case reports have documented presence of paediatric rheumatic disorders in Tanzania. This study was conducted to determine the spectrum of rheumatic disorders among children at Muhimbili National Hospital (MNH).

**Methods:**

This was a retrospective study conducted among children who were attended at MNH between January 2012 and August 2019. Paediatric patients seen in the out-patient clinics and those admitted in the wards were eligible. All patients with diagnosis of rheumatic disorders were identified from admission books and outpatient clinic logbooks, and later data were collected from their case notes and were recorded in clinical research forms. Collected information included age, sex, clinical features and laboratory tests results.

**Results:**

A total of 52 children with mean age of 9.5 ± 4.3 years, 12 (40.4%) participants were aged above 10 years and 32 (61.5%) were females. Frequently reported clinical presentations were joint pain 44 (84.6%), joint swelling 34 (65.4%), fever 24 (46.2%) and skin rashes 21(40.4%). Juvenile idiopathic arthritis (JIA) was the predominant diagnosis reported in 28 (53.8%) participants followed by juvenile systemic lupus erythematosus 8 (15.4%), mixed connective tissue diseases 4 (7.7%) and juvenile dermatomyositis 4 (7.7%). Antinuclear antibody test was performed in 16 participants it was positive in 9 (56.2%). Nine participants were tested for anti-double stranded DNA test and 5 (55.6%) were positive for this test. C-reactive protein was tested in 46 participants out of which 32 (69.6%) had elevated levels. HIV was tested in 24 (46.2%) participants and results were negative. Thirty-five out of 52 (67.3%) participants had anaemia. Predominant drugs used for treatment of JIA include prednisolone and methotrexate.

**Conclusions:**

Paediatric rheumatic disorders are not uncommon in Tanzania-and were noted to affect more female children in this study. Predominant conditions included juvenile idiopathic arthritis (JIA), juvenile systemic lupus erythematosus (JSLE) and juvenile dermatomyositis (JDM).

## Background

Non-communicable diseases (NCD) have assumed significant contribution in the morbidity and mortality globally [1], this has resulted in the strain in the health systems in lower income countries especially those in sub-Saharan Africa which have been struggling with infectious diseases. In Tanzania similar trend of increasing burden of NCD has been documented [2]. Musculoskeletal conditions which result in long term disability, pain and poor quality of life are reported to have high global burden which prompted a decade of bone and joint campaign (2000–2010) established jointly by European League Against Rheumatism (EULAR) and World Health Organization (WHO) with the aim of creating awareness about musculoskeletal conditions globally [3, 4].

There has been a long-standing myth that musculoskeletal disorders are rare in Africa and this myth has been partly supported by limited capacity to diagnose these conditions and lack of health care providers’ awareness [5, 6]. There is limited information about the magnitude of these conditions in Sub-Saharan Africa (SSA) which is home to more than 350 million children [5]. Few studies and case reports from this region have documented common occurrence of these conditions especially for adult population [6].

Rheumatic disorders accounted for 0.32% of all paediatric admission in 2011 at Getrude’s Children’s Hospital in Nairobi, Kenya as reported by Migowa et al. [7] The commonest rheumatic condition reported was inflammatory arthropathy which accounted for 42.3%, other conditions documented included septic arthritis, Kawasaki disease and rheumatic fever [7].

A retrospective review of paediatric rheumatic disorders for 5 years conducted at Lagos State University Teaching Hospital by Olaosebikan et al., revealed juvenile idiopathic arthritis as the commonest paediatric rheumatic disorder accounting for 49.1%, other rheumatic conditions noted in that review included juvenile systemic lupus erythematosus, juvenile systemic sclerosis, juvenile dermatomyositis, fibromyalgia and hypermobility syndrome [8]. The spectrum of rheumatic conditions reported in that review illustrates the prevalence of these conditions in children of SSA.

Juvenile idiopathic arthritis is reported as the most common paediatric rheumatic disorder globally [9]. Several studies conducted in SSA have described this condition in children; Chipeta et al. described 78 children with JIA in a retrospective study conducted at University Teaching Hospital, Lusaka Zambia, of these 43 were females and 35 were males and an average age at disease onset of 8.7 years [10].

Two case reports have been published documenting existence of paediatric rheumatic conditions in Tanzania. Noorani et al. described two cases of Kawasaki Diseases who were managed at one of hospital in Tanzania while Grijsen et al. described a four-year-old girl who presented with dermatomyositis [11, 12]. The lack of information on the burden of rheumatic disorders among children in Tanzania prompted this study to be carried out at Muhimbili National Hospital.

## Methods

### Study design and study area

This was a retrospective chart review of all children with rheumatic disorders who received services in the paediatric department at Muhimbili National Hospital (MNH) between January 2012 and August 2019.These patients were either admitted in paediatric wards or attended outpatient paediatric clinics. There is no paediatric rheumatology clinic at MNH, however since early 2016 patients with paediatric rheumatic conditions have been seen in paediatric nephrology clinic at MNH. Since early 2016 children with rheumatic disorders have been attended by a paediatric nephrologist who has been trained in paediatric rheumatology through European League Against Rheumatism (EULAR)/Paediatric Rheumatology European Society (PReS) online training and with clinical apprenticeship at Red Cross Children Hospital. This study was carried out among children aged 0–17 years who received services at MNH between January 2012 and August 2019. Muhimbili National Hospital is the national referral hospital for Tanzania and it also serve as tertiary referral hospital for three regional hospitals in Dar es Salaam city which has a population of about 8 million people. It is the teaching hospital for Muhimbili University of Health and Allied Sciences. All participants recruited in this study were referred from regional and referral hospitals in Tanzania.

### Sampling technique

Patients with rheumatic diseases who were treated at MNH (as in patient or outpatient clinic) between January 2012 and august 2019 were identified though admission books and outpatient attendance books.

### Data collection

Data was collected from participants’ case notes and were recorded into clinical research forms (CRF). Information which was collected included age, sex, diagnosis, and laboratory tests results. The diagnoses criteria utilized for participants recruited in this study included American College of Rheumatism criteria for juvenile systemic erythematosus and Sjogren syndrome, International League Against Rheumatism for juvenile idiopathic arthritis and European League Against Rheumatism/ American College of Rheumatism criteria for juvenile dermatomysoistis.

### Data analysis

All filled CRFs were coded before entering Statistical Package for Social Sciences version 22. Data cleaning and analysis was performed using the same SPSS version 22. Data were summarized into frequency distribution tables.

### Ethical consideration

Ethical clearance was sought from MUHAS Institutional Review Board and permission to conduct the study at the national hospital was granted by administration. A waiver for consent was requested from MUHAS IRB as this was a retrospective chart review. No information that could identify participants was collected and each participant was assigned a unique identification number which was linked with hospital registration number to avoid double recruitment. All information collected was kept as confidential.

## Results

### Demographic and clinical features of participants

Fifty-two children were identified and recruited into this study. Mean age of study participants was 9.5 ± 4.3 years, 12 (40.4%) were aged above 10 years and 32 (61.5%) were female. The most common clinical presentation of study participants was joint pain which was noted in 44 (84.6%) of participants followed by joint swelling 34(65.4%), fever24 (46.2%) and skin rashes 21(40.4%), Table 1.

**Table 1:** Demographic and clinical features of participants

Variable	Number (%)
**Age (years)**	
1–5	12 (23.1)
> 5–10	19 (36.5)
> 10	21 (40.4)
**Sex**	
Male	20 (38.5)
Female	32 (61.5)
**Clinical features**	
Fever	24 (46.2)
Joint pain	44 (84.6)
Joint swelling	34 (65.4)
Bone pain	1 (1.9)
Skin rash	21 (40.4)
Muscle pain	4 (7.7)
Muscle weakness	5 (9.6)
Dysphagia	4 (7.7)
Dysphonia	2 (3.8)
Dry eyes	1 (1.9)
Dry mouth	1 (1.9)
Headache	2 (3.8)
Photosensitivity	7 (13.5)
Alopecia	9 (17.3)
Skin ulcers	2 (3.8)
Digital gangrene	1 (1.9)
Weight loss	9 (17.3)
Lymphadenopathy	7 (13.5)
Calcinosis	1 (1.9)
Gottron’s papule	4 (7.7)
Periorbital oedema	4 (7.7)
Absent peripheral pulses	1 (1.9)
Haematuria	1 (1.9)

### Rheumatologic diagnoses

Table 2 describe rheumatologic diagnoses which were noted in this study showing juvenile idiopathic arthritis (JIA) as the predominant diagnosis noted among 28 (53.8%) participants. Among those with JIA 21 (75%), 6 (21.4%) and 1 (3.6%) had polyarticular, systemic and oligoarticular types of JIA respectively. The other diagnoses which were noted included systemic lupus erythematosus 8 (15.4%), mixed connective tissue diseases 4 (7.7%) and juvenile dermatomyositis 4 (7.7%). Out of the two participants with other vasculitis one had IgA vasculitis. More than half of participants with JIA were females and all participants with SLE were females.

**Table 2:** Rheumatologic diagnoses

Diagnosis	Total Number (%)	Gender distribution	
		M (%)	F (%)
Juvenile idiopathic arthritis (JIA)	28 (53.8)	13 (46.5)	15 (53.5)
Juvenile Systemic Lupus Erythematosus (JSLE)	8 (15.4)	0 (0)	8 (100)
Mixed Connective Tissue Disease (MCTD)	4 (7.7)	2 (50)	2 (50)
Juvenile Dermatomyositis (JDM)	4 (7.7)	0 (0)	4 (100)
Takayasu Arteritis	2 (3.8)	2 (100)	0 (0)
Kawasaki Disease	2 (3.8)	2 (100)	0 (0)
Other vasculiti	2 (3.8)	1 (50)	1 (50)
Sjogren Disease	1 (1.9)	0 (0)	1 (100)
Chronic Recurrent Multifocal Osteomyelitis (CRMO)	1 (1.9)	0 (0)	1 (100)

### Laboratory tests performed for participants

Tables 3 and 4 describe laboratory test results for study participants, these test results were not available for all participants. Sixteen participants had Antinuclear antibody test done out of which nine (56.2%) were positive, for anti-double stranded DNA tests five (55.6%) were positive out of 9 tests. C-reactive protein (CRP) was available for 46 participants out of which 32 (69.6%) elevated for which 19 (76%) out of 25 participants who had CRP tested had elevated levels. Human Immunodeficiency Virus (HIV) was test was performed in 24 participants and all results were negative and 35 (67.3%) participants had anaemia.

**Table 3:** Participants’ laboratory test results

Laboratory tests	Number (%) / (Min-max)
**Antinuclear Antibody**	
Positive	9 (52.9)
Negative	8 (47.1)
**Anti-cytoplasmic antibody**	
Negative	4 (100)
**Anti dsDNA antibody**	
Positive	5 (55.6)
Negative	4 (44.4)
**Anti-Smith Antibody**	
Positive	1 (50)
Negative	1 (50)
**Anti RNP antibody**	
Positive	1 (50)
Negative	1 (50)
**Complement C3 level**	
Normal	5 (83.3)
Low	1 (16.7)
**Complement C4 level**	
Normal	5 (83.3)
Low	1 (16.7)
**C-Reactive Protein**	
1–5 ng/ml	14 (30.4)
> 5 ng/ml and above	32 (69.6)
**Erythrocyte Sedimentation Rate**	
1–20 mm in the first hour	6 (15)
> 20	34 (85)
**Haemoglobin level (d/dL)**	
< 11	35 (67.3)
11 and above	17 (31.4)
**HIV antibody test**	
Negative	24 (100)
**Hepatitis B sAg**	
Negative	22 (100)
**Hepatitis C antibody**	
Negative	22 (100)
**Haemoglobin**	3.1–15.4
**White blood cell count**	1.2–24.1
**Platelet count**	60–1412
**C-reactive Protein**	0.3–425
**Erythrocyte sedimentation rate**	7–160
**Serum creatinine**	24–1781
**Alanine aminotransferase**	6–165
**Aspartate aminotransferase**	8–321
**Lactate dehydrogenase**	163–1498
**Creatinine kinase**	21–4268
**Ferritin**	17.5–2000

**Table 4:** Participants’ diagnoses and CRP and ANA results

Diagnosis	CRP		ANA	
	1–5 mg/dL n (%)	> 5 mg/dL n (%)	Positive n (%)	Negative n (%)
JIA	6 (24)	19 (76%)	0 (0)	3 (100)
JSLE	3 (42.8)	4 (57.2)	8 (100)	0
MCTD	1 (33.3)	2 (66.7)	1 (33.3)	2 (67.3)
Kawasaki disease	0 (0)	2 (100)	–	–
Takayasu arteritis	1 (50)	1 (50)	–	–
JDM	2 (66.7)	1 (33.3)	0 (0)	1 (100)
Sjogren syndrome	0 (0)	1 (100)	1 (100)	0 (0)
CRMO	1 (100)	0 (0)	–	–
Other vasculitis	0 (0)	2 (100)	0 (0)	1 (100)

**Fig. 1:**
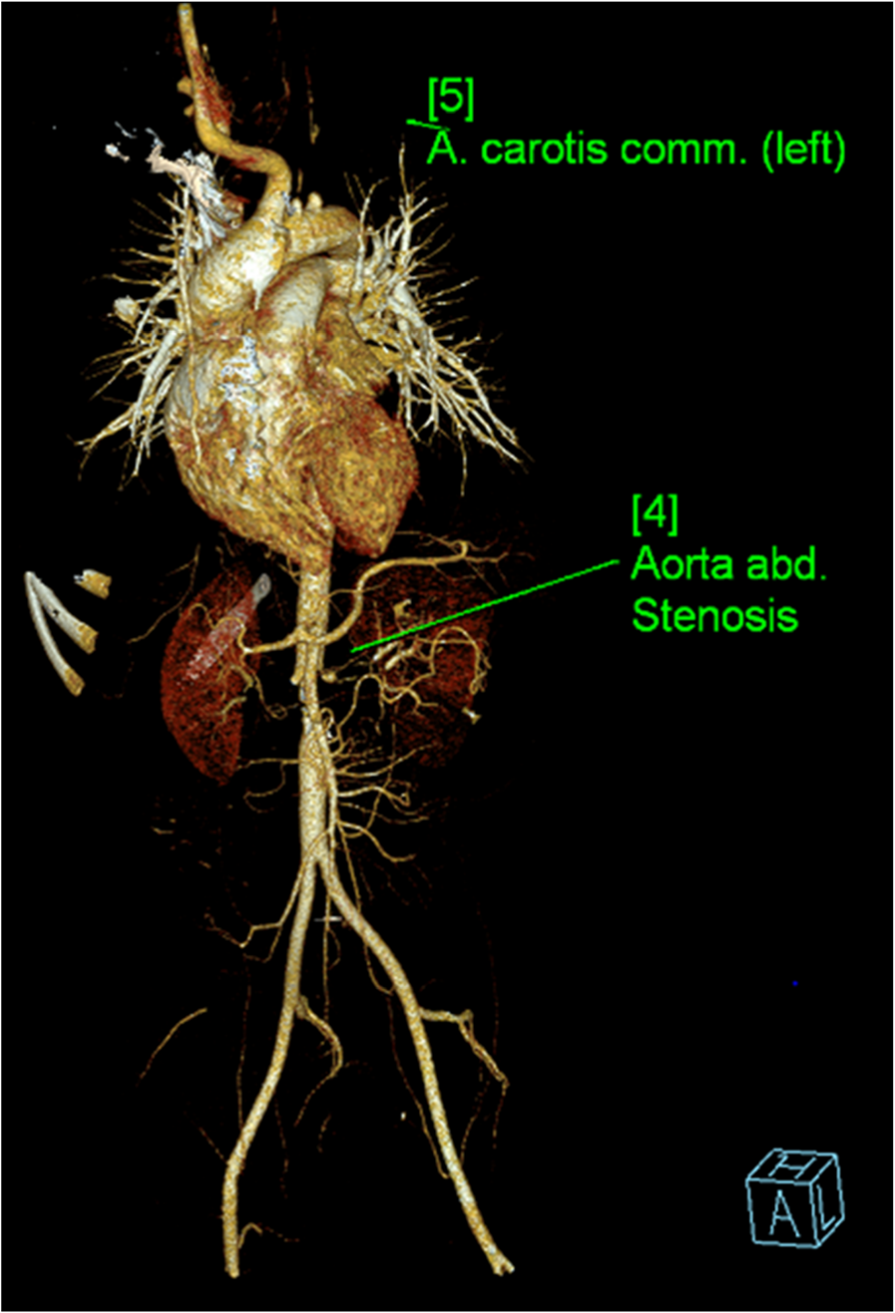
Computerized Tomographic Aortogram of a participant with Takayasu arteritis

**Fig. 2:**
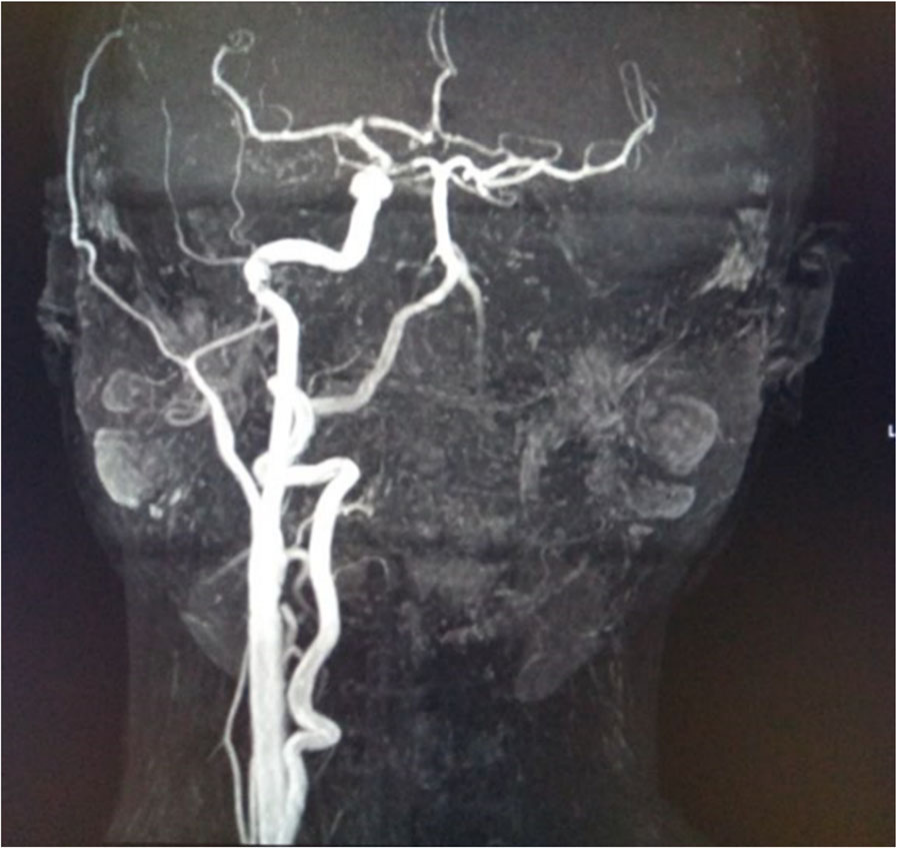
Magnetic Resonance Angiogram of a patient with Takayasu arteritis

Echocardiogram were performed for participants with Kawasaki Disease showing normal findings in one participant and dilated coronary artery for the other. Out of the Computerized Tomographic aortogram scan and Magnetic resonance angiogram were performed for participants with Takayasu arteritis. In one participant narrowing of the thoracic and abdominal aorta and severe narrowing of both renal arteries were noted. For the other participant narrowing of thoracic and abdominal aorta and involvement of the left common carotid artery with no visualization of left common carotid artery, left internal and external carotid arteries and left vertebral artery were noted, Figs. 1 and 2.

### Treatment of patients with paediatric rheumatic diseases

All patients with JIA except few with oligoarticular JIA were treated with prednisolone initially and later given Methotrexate, one patient with oligoarticular JIA was treated with non-steroid anti-inflammatory drugs (NSAIDS) only. Three patients were not responding with methotrexate, one received Tocilizumab, one was given combination of methotrexate and leflunomide and one had combination of methotrexate and sulfasalazine. One patient with JIA had prolonged use of high dose steroids and suffered avascular necrosis neck of femur and she has undergone bilateral hip replacement surgery.

Two participants with JSLE had lupus nephritis one had renal biopsy done showing class III lupus nephritis, the other was not biopsied, they were treated with prednisolone (initially with methylprednisolone), cyclophosphamide, and for continuation phase one was given azathioprine and the other mycophenolate mofetil. One patient was receiving hydroxychloroquine and prednisolone. Other patients were treated with prednisolone.

The two patients with Takayasu arteritis received prednisolone (initially with methylprednisolone) one dose of cyclophosphamide and methotrexate. Patients with Kawasaki diseases were treated with intravenous Immunoglobulin (IVIG) while those with mixed connective tissue disease (MCTD) were treated with prednisolone. Participant with chronic recurrent multi-focal osteomyelitis (CRMO) was given non steroid anti-inflammatory drugs (NSAIDS).

Four (50%) participants with Juvenile SLE and two participants with juvenile dermatomyositis (50%) had died at the time of accessing the charts.

## Discussion

There is dearth of information regarding rheumatic disorders in lower income countries particularly in the sub-Saharan region, this scarcity of data is even more severe for paediatric rheumatic disorders. Fifty-two children who presented with rheumatic disorder to the paediatric department at Muhimbili National Hospital in Dar es Salaam, Tanzania were identified in this retrospective study. Thirty-two (61.5%) out of 52 children with rheumatic disorder were females and the mean age at presentation was 9.5 ± 4.3 years.

Presentation age noted in our study is lower than what was reported in a study conducted among Indian children reporting mean age at presentation of 11 ± 3.3 years, age at onset of symptoms for Indian children was reported to be 9.1 ± 3.6 years [13]. Olaosebikan et al. reported much older age at presentation (14 ± 4.4 vs 9.5 ± 4.3), which might be attributed to the setting of their study which was the adult rheumatology clinic [8]. Late presentation is common in developing countries which have poorly established paediatric rheumatology services, this is depicted by the older mean age at presentation noted in our study and those from India and Nigeria as compared to studies from developed countries like US [14].

Predominance of female children was observed in our study, this is like report by Olaosebikan et al. from Nigeria, this similarity is partly attributed to the predominant rheumatic disorders note in the two reports whereby juvenile idiopathic arthritis was the most frequent condition [8]. Migowa et al. and Patra et al. reported predominance of male children in their reports, of interest is the fact that Patra et al. observed predominance of JIA as was a case for our findings, while Migowa et al. did not document JIA [7, 13].

Consistent with reports from other studies on rheumatic disorders in children joint pain was the most frequent clinical feature noted in this study, musculoskeletal pain was reported in 91.2% of children with rheumatic disorders in Nigeria which is close to our report of 84.6% [8]. Constitutional symptoms also featured in this study and highlighting the importance of considering paediatric rheumatic disorders as possible differentials for children with this presentation [8, 15]. Fever was noted as presenting feature in 46% of participants, this might not be attributed entirely to rheumatic conditions, since malaria and other tropical infectious conditions are common in Tanzania.

The most frequent rheumatic disorder noted in our study was JIA which accounted for 54% followed by JSLE 15% then MCTD and JDM each accounting for.

7%. This result support previous reports in sub-Saharan region with predominance of JIA, as was reported by Okong’o et al. and Olaosebikan et al. in studies conducted among children in Cape Town, South Africa and Nigeria respectively [8, 16]. Similar findings were reported from studies conducted in other regions of the world as described in India, Austria and Yemen [13, 17]. Majority of participants with JIA had polyarticular form, which is similar to findings by Weakley et al. from a study conducted in Cape Town, South Africa, this may reflect the fact that children who were referred were those who were very sick [18].

Mixed connective tissue disease (MCTD), Kawasaki disease, Takayasu arteritis, vasculitis, Sjogren syndrome and CRMO featured in this study. One of the two participants with vasculitis had IgA vasculitis. From these findings it is evident that different paediatric rheumatic conditions are common Tanzania, this was also depicted in the previous case reports from Tanzania [11, 12]. The pattern of rheumatic disorders presented by this study break the myths that these conditions are not existing in the region, and in fact some conditions like SLE are reported to be more common among people of African descent [19].

Four participants had juvenile dermatomyositis in this study, this is one of the very rare connective tissue disorders, whose incidence is estimated to be 2–4 children per million population [20, 21]. Two of these participants were dead at the time of collecting data as was noted from the charts. Diagnosing dermatomyositis is usually challenging and rely on clinical criteria with no known confirmatory diagnostic tests requiring good clinical acumen [22]. Dermatomyositis has been described among African children as was noted by Faller et al. and Okonkwo et al. in Johannesburg and Cape Town respectively [23, 24]. Patients with JDM may also present to neurologists and dermatologists because of various patterns of manifestations [25].

Kawasaki disease, Takayasu arteritis and IgA vasculitis were observed in this study, our findings which are consistent with reports from India, South Africa and Kenya and demonstrate existence of these rare rheumatic disorder in Tanzania [7, 13, 16, 26]. Kawasaki disease has been previously described from Tanzania in a case report by Noorani et al. [11] Interesting Olaosebikan et al. did not find any case of vasculitis in a study conducted in Nigeria [8].

Laboratory tests were performed for participants enrolled in this study including autoimmune markers which were taken to private laboratories. Viral screening for Human Immunodeficiency Virus (HIV), Hepatitis B virus and Hepatitis C virus were carried out for 24 (46.2%), 22 (42.3%) and 22 (42.3%) participants respectively and were all negative. HIV prevalence in Tanzania is estimated to be 5.5%, and HIV testing is advocated for patients at risk [27]. more than half of participants were not tested for HIV. Testing for HIV is important as arthritis has been reported to be one of manifestation of HIV infection in children [28]. The lowest haemoglobin level observed in this study was 3.1 g/dL and 35 (67.3%) out of all participants had anaemia. Several factors could have contributed to the noted burden of anaemia including worm infestation, dietary deficiency and chronic inflammation.

Tests for determining disease activity were also performed in this study, these included erythrocyte sedimentation rate (ESR), C-reactive protein (CRP), alanine aminotransferase (ALT), aspartate aminotransferase (AST), creatinine kinase (CK) and lactate dehydrogenase (LDH). Imaging studies were utilized for evaluating participants with Kawasaki diseases and Takayasu arteritis. Typical findings of Takayasu arteritis were described in this study with involvement of aorta and its branches consistent with report by McCulloch et al. [25]

Treatment of paediatric rheumatic conditions is reported to be challenging in developing countries lower income countries because of difficulties in diagnosis, late presentation as well as limited option with medications [6, 9, 26]. .Patients with JIA in this study were treated mainly with prednisolone and methotrexate. Refractory JIA was treated with combination of methotrexate with leflunomide or sulfasalazine. Only on patient was noted to have used biologic tocilizumab for treatment of JIA which was used for a short duration of 4 months. These drugs are not available in the country, so parents had to buy them from overseas. One participant with JIA had bilateral hip replacement surgery following complication of long-term use of steroids.

There are unmet needs of care for children with rheumatic disorders in Tanzania as reflected by high mortality for JSLE and JDM and severe associated morbidity of children recruited in this study as depicted by high burden of anaemia. Paediatric rheumatology services introduced by the paediatric nephrologist at MNH is a positive step in establishing proper care for children in Tanzania and there is a need to support this initiative with short term training for primary care physicians and general paediatrician so that they can identify children with rheumatic disorders timely and prevent complications and mortality [9, 29].

This study was conducted at Muhimbili National Hospital, which is the national referral hospital, therefore these findings might provide reasonable baseline data for Tanzania. However, it is possible that the study setting might have missed children who presented to other departments including dermatology and orthopaedics, some children could have been misdiagnosed and other might have died before being referred to MNH.

## Conclusions

Rheumatic disorders are not uncommon among children in Tanzania as described in this study, the predominant conditions noted were juvenile idiopathic arthritis (JIA) followed by juvenile systemic lupus erythematosus (JSLE) and juvenile dermatomyositis (JDM). Female children were more affected than male counterparts. Laboratory testing and imaging were performed for diagnosing and assessing diseases activity for children with rheumatic disorders and these including ESR, CRP, ALT, AST, LDH and autoimmune markers. Radiological imaging included echocardiogram for diagnosing Kawasaki diseases and magnetic resonance imaging and computerized tomographic scans for Takayasu arteritis.

## Data Availability

The datasets used and/or analysed during the current study are available from the corresponding author on reasonable request.

## Abbreviations

ALT: Alanine aminotransferase
AST: Aspartate aminotransferase
CK: Creatinine kinase
CRF: Clinical research form
CRMO: Chronic recurrent multifocal osteomyelitis
CRP: C-reactive protein
EULAR: European League Against Rheumatism
HIV: Human Immunodeficiency Virus
IRB: Institutional Review Board
IVIG: Intravenous immunoglobulin
JDM: Juvenile dermatomyositis
JIA: Juvenile idiopathic arthritis
JSLE: Juvenile systemic lupus erythematosus
LDH: Lactate Dehydrogenase
MCTD: Mixed connective tissue disease
MNH: Muhimbili National Hospital
MUHAS: Muhimbili University of Health and Allied Sciences
NCD: Non-communicable disease
NSAID: Non steroid anti-inflammatory drugs
PReS: Paediatric Rheumatology European Society
SPSS: Statistical Package for Social Sciences
SSA: Sub-Saharan Africa
US: United States
WHO: World Health Organization

## References

[1] Naghavi M, Abajobir AA, Abbafati C (2017). Global, regional and national age-sex specific mortality for 264 causes of death, 1980–2016: a systematic analysis for the global burden of disease study 2016. Lancet.

[2] Mayige M, Kagaruki G, Ramaiya K, Swai A (2012). Non communicable diseases in Tanzania: a call for urgent action. Tanzania J Heal Res.

[3] Woolf AD, Pfleger B (2003). Burden of major musculoskeletal conditions. Bull World Health Organ.

[4] Woolf AD (2000). The bone and joint decade 2000–2010. Ann Rheum Dis.

[5] Scott C, Webb K (2014). Paediatric rheumatology in sub-Saharan Africa. Rheumatology.

[6] Mody GM (2017). Rheumatology in Africa—challenges and opportunities. Arthritis Res Ther.

[7] Migowa A, Colmegna I, Hitchon C, Were E, Ng’ang’a E, Ngwiri T, Wachira J, Bernatsky S, Scuccimarri R (2017). The spectrum of rheumatic in-patient diagnoses at a pediatric hospital in Kenya. Pediatr Rheumatol.

[8] Olaosebikan BH, Adelowo OO, Animashaun BA, Akintayo RO (2017). Spectrum of paediatric rheumatic diseases in Nigeria. Pediatr Rheumatol.

[9] Scott C, Chan M, Slamang W, Okong’o L, Petty R, Laxer RM, Katsicas M, et al. Juvenile arthritis management in less resourced countries (JAMLess): consensus recommendations from the cradle of humankind. Clin Rheumatol. 2018. 10.1007/s10067-018-4304-y [Epub ahead of print]. 10.1007/s10067-018-4304-y 30267356

[10] Chipeta J, Njobvu P, Wa-Somwe S, Chintu C, McGill PE, Bucala R (2013). Clinical patterns of juvenile idiopathic arthritis in Zambia. Pediatr Rheumatol.

[11] Noorani M, Lakhani N (2018). Kawasaki disease: two case reports from the Aga Khan Hospital, Dar es Salaam-Tanzania. BMC Pediatr.

[12] Grijsen ML, Mchaile D, Geut I, Olomi R, Nwako M, Requena L, Howlett WP, Mavura DR, Dekker MCJ (2017). Juvenile dermatomyositis in a 4-year-old Kenyan girl. Clin Case Rep.

[13] Patra PK, Kumar M (2018). Clinico-epidemiological profile of pediatric rheumatology disorders in eastern India. J Nat Sci Biol Med.

[14] Mahajan M, Shah M, Toth M, Mcninch N, El-Hallak M (2014). Inpatient pediatric rheumatic diseases: characteristics, cost and trends. Arthritis Rheum.

[15] Dahman HA (2017). Challenges in the diagnosis and management of pediatric rheumatology in the developing world: lessons from a newly established clinic in Yemen. Sudan J Paediatr.

[16] Okong’o LO, Scott C (2014). The spectrum of paediatric rheumatic diseases in two tertiary centres in Cape Town, South Africa. Pediatr Rheumatol.

[17] Huemer C, Huemer M, Dorner T, Falger J, Schacherl H, Bernecker M, Artacker G, Pilz I (2001). Incidence of pediatric rheumatic diseases in a regional population in Austria. J Rheumatol.

[18] Weakley K, Esser M, Scott C (2012). Juvenile idiopathic arthritis in two tertiary centres in the Western Cape, South Africa. Pediatr Rheumatol.

[19] Scott C, Webb K (2014). Paediatric rheumatology in sub-Saharan Africa-time to narrow the gap. Rheumatology.

[20] Oddis CV, Conte CG, Steen VD, Medsger TA (1990). Incidence of polymyositis – dermatomyositis: a 20- year study of hospital diagnosed cases in Allegheny Country, PA 1963–1982. J Rheumatol.

[21] Pelkonen PM, Jalanko HJ, Lantto RK, Makela AL, Pietikainen MA, Savolainen HA, Verronen PM (1994). Incidence of systemic connective tissue diseases in children: a nationwide prospective study in Finland. J Rheumatol.

[22] Hinze CH, Oommen PT, Dressler F, Urban A, Weller-Heinemann F, Speth F, Lainka E, Brunner J, Fesq H, Foell D, Müller-Felber W, Neudorf U, Rietschel C, Schwarz T, Schara U, Haas J (2018). Development of practice and consensus based strategies including a treat-to-target approach for the management of moderate and severe juvenile dermatomyositis in Germany and Austria. Pediatr Rheumatol.

[23] Faller G, Mistry BJ, Tikly M (2014). Juvenile dermatomyositis in south African children is characterised by frequent dystropic calcification: a cross sectional study. Pediatr Rheumatol.

[24] Okong’o LO, Esser M, Wilmshurst J, Scott C (2016). Characteristics and outcome of children with juvenile dermatomyositis in Cape Town: a cross-sectional study. Pediatr Rheumatol.

[25] Hilton-Jones D (2003). Diagnosis and treatment of inflammatory muscle diseases. J Neurol Neurosurg Psychiatry.

[26] McCulloch M, Andronikou S, Goddard E, Sinclair P, Lawrenson J, Mandelstam S, Beningfield SJ, Millar AJ (2003). Angiographic features of 26 children with Takayasu’s arteritis. Pediatr Radiol.

[27] Ministry of Health, Community Development, Gender, Elderly and Children (MoHCDGEC) and the Ministry of Health (MoH) Zanzibar (2017). Tanzania HIV impact survey (THIS) 2016–2017: preliminary findings.

[28] Chinniah K, Mody GM, Bhimma R, Adhikari M (2005). Arthritis in association with human immunodeficiency virus infection in Black African children: cause or coincidental?. Rheumatology.

[29] Al Maini M, Adelowo F, Al Saleh J, Al Weshahi Y, Burmester G, Cutolo M, Flood J, March L, McDonald-Blumer H, Pile K, Pineda C, Thorne C, Kvien TK (2015). The global challenges and opportunities in the practice of rheumatology: white paper by the world forum on rheumatic and musculoskeletal diseases. Clin Rheumatol.

